# Exploring the Utility of Community-Generated Social Media Content for Detecting Depression: An Analytical Study on Instagram

**DOI:** 10.2196/11817

**Published:** 2018-12-06

**Authors:** Benjamin J Ricard, Lisa A Marsch, Benjamin Crosier, Saeed Hassanpour

**Affiliations:** 1 Department of Biomedical Data Science Dartmouth College Lebanon, NH United States; 2 Center for Technology and Behavioral Health Dartmouth College Hanover, NH United States; 3 Department of Psychiatry Dartmouth College Hanover, NH United States; 4 Department of Epidemiology Dartmouth College Hanover, NH United States; 5 Department of Computer Science Dartmouth College Hanover, NH United States

**Keywords:** machine learning, depression, social media, mental health

## Abstract

**Background:**

The content produced by individuals on various social media platforms has been successfully used to identify mental illness, including depression. However, most of the previous work in this area has focused on *user-generated* content, that is, content created by the individual, such as an individual’s posts and pictures. In this study, we explored the predictive capability of *community-generated* content, that is, the data generated by a community of friends or followers, rather than by a sole individual, to identify depression among social media users.

**Objective:**

The objective of this research was to evaluate the utility of community-generated content on social media, such as comments on an individual’s posts, to predict depression as defined by the clinically validated Patient Health Questionnaire-8 (PHQ-8) assessment questionnaire. We hypothesized that the results of this research may provide new insights into next generation of population-level mental illness risk assessment and intervention delivery.

**Methods:**

We created a Web-based survey on a crowdsourcing platform through which participants granted access to their Instagram profiles as well as provided their responses to PHQ-8 as a reference standard for depression status. After data quality assurance and postprocessing, the study analyzed the data of 749 participants. To build our predictive model, linguistic features were extracted from Instagram post captions and comments, including multiple sentiment scores, emoji sentiment analysis results, and meta-variables such as the number of likes and average comment length. In this study, 10.4% (78/749) of the data were held out as a test set. The remaining 89.6% (671/749) of the data were used to train an elastic-net regularized linear regression model to predict PHQ-8 scores. We compared different versions of this model (ie, a model trained on only user-generated data, a model trained on only community-generated data, and a model trained on the combination of both types of data) on a test set to explore the utility of community-generated data in our predictive analysis.

**Results:**

The 2 models, the first trained on only community-generated data (area under curve [AUC]=0.71) and the second trained on a combination of user-generated and community-generated data (AUC=0.72), had statistically significant performances for predicting depression based on the Mann-Whitney *U* test (*P=*.03 and *P=*.02, respectively). The model trained on only user-generated data (AUC=0.63; *P=*.11) did not achieve statistically significant results. The coefficients of the models revealed that our combined data classifier effectively amalgamated both user-generated and community-generated data and that the 2 feature sets were complementary and contained nonoverlapping information in our predictive analysis.

**Conclusions:**

The results presented in this study indicate that leveraging community-generated data from social media, in addition to user-generated data, can be informative for predicting depression among social media users.

## Introduction

### Background

Major depressive disorder (MDD) is estimated to be the second leading cause of disability burden worldwide and can contribute to a variety of health complications, particularly contributing to increased rates of suicide and ischemic heart disease [1]. However, many cases of MDD remain untreated due to the difficulty in identifying the disease: nonpsychiatric physicians diagnose depression in less than half of their patients with MDD, even with 5 years of follow-up care [2]. The United States Preventative Services Task Force recommends depression screening in the general adult population, particularly in pregnant and postpartum women [3]. Screening in hospital settings and medical practices may aid providers in identifying depression; however, screening methods need to be efficiently and feasibly implemented in medical care settings [4,5]. In addition to the promise of providing information about patients at risk of MDD, these methods may also be applicable to other mental illnesses, such as drug addiction [6,7]. Furthermore, social media’s potential causal effects on depression may be controlled for by exploring this data source’s predictive capability [8].

Social media content may be useful in expanding efforts to identify mental disorders at a population level and in facilitating the delivery of interventions to otherwise undiagnosed social media users. Designing a mental health screening methodology using social media data offers the potential to reach a broad population, including lower-income and minority individuals who may be undiagnosed and untreated for MDD, as teenagers and adults use social media in comparable levels across these socioeconomic and demographic groups [8-12]. As of May 2018, 35% of US adults use Instagram, an online social media platform for users to share pictures and video, including 64% of individuals aged 18 to 29 years [9]. Instagram posts consist of a photo or video and, optionally, a caption provided by the user and comment and likes from other users. Instagram photos have been shown to contain information relevant to depression status [10].

### Objectives

Recent evidence shows the significant predictive power of social media to identify MDD, particularly in users of Twitter and Facebook [13-16]. Reece and Danforth created a model trained on signals indicative of depression in Instagram posts, such as the number of comments and the color of images, to predict depression as measured by the Center for Epidemiologic Studies Depression Scale [13]. Word-based score approaches, where each word corresponds to a specific score, have been shown to contain information regarding depression. Specifically, Affective Norms for English Words (ANEW) and Language assessment by Mechanical Turk (LabMT) were used to predict depression among Twitter users [14]. Previous work has also established the ability of Facebook status updates to predict postpartum depression as measured using the Patient Health Questionnaire (PHQ) [15]. Other studies indicate that depression in users of Twitter and Facebook could be identified through word sentiment analysis, particularly with regard to daily variation of sentiment in users, as well as through the use of metadata [16,17].

The majority of research conducted to predict MDD in social media users has focused on *user-generated* content, that is, the content created by the users themselves. This includes Twitter “Tweets,” Facebook status updates, and images/videos created by the user and subsequently shared with their peers. The other information available for a given user is *community-generated* content, such as a post’s “likes”/comments, friends’ “wall” posts, and followers, all of which are not generated by individual users themselves but contain information on a given user and friend pair’s bidirectional engagement on a social media platform. In this study, we hypothesized that word-based community-generated content contains information that can be utilized for MDD screening. We also aimed to directly test if user-generated and community-generated content contain complementary information indicative of an individual’s MDD status.

## Methods

### Recruitment

The Clickworker crowdsourcing platform was used to recruit study participants. This platform is similar to Amazon’s Mechanical Turk; however, its policy on sharing social media content on the platform was more suitable for this study. Participants’ time completing surveys was compensated via monetary payment. Following consent, participants were asked to respond to survey questions, including the PHQ-8 questionnaire responses, and provide access to their Instagram profiles. Instagram profiles consist of a series of posts (an example Instagram post is shown in Multimedia Appendix 1). These posts can have captions, which are written by the user, and comments, which are mostly written by the user’s followers. The Instagram application programming interface was used to automatically mine relevant features, with “/users/self,” “/users/self/media/recent,” and “/media/{media-id}/comments” as the end points. This data collection, the study’s methodology, and the use of data in our study were approved by the Dartmouth Institutional Review Board. The research presented in this paper was conducted with participants’ informed consent and complies with the World Medical Association Declaration of Helsinki on Ethical Principles for Medical Research Involving Human Subjects.

#### Patient Health Questionnaire-8 Mental Health Questionnaire

To quantify MDD in our study, PHQ-8 was completed by Instagram users. This 8-question inventory surveys the incidence of MDD symptoms over the prior 2 weeks and was created according to the Diagnostic and Statistical Manual of Mental Disorders, 4th Edition criteria for depression over the prior 2 weeks. The PHQ-8 is identical in content and scoring to the PHQ-9; however, it does not include the last question of the PHQ-9 regarding suicidal/self-injury thoughts, as previous studies have shown this question does not provide significant additional information regarding MDD risk [18]. For each symptom, the respondent is asked to identify whether they felt each symptom, for example, “Feeling down, depressed, or hopeless,” and to select how often they felt each symptom: not at all (value of “0”), several days (“1”), more than half the days (“2”), and nearly every day (“3”) over the past 2 weeks. The full PHQ-8 questionnaire is included in Multimedia Appendix 2. These values were added together to create a composite score between “0” and “24” for each user. A score above “10” usually signifies MDD; however, the scores between “10” and “14” have been called a “gray zone” in which some individuals are false positives for MDD, whereas a score above “15” is strongly indicative of MDD [19,20]. For the purposes of this study, we defined MDD using a PHQ-8 score cutoff of “15,” following the example of a previous study that used the same cutoff to analyze postpartum depression in Facebook users [21].

### Sentiment Analysis

Replicating the variable selection protocol from a previous study [14], information was extracted from the texts of post captions and comments using 3 unigram frequency–based approaches: ANEW, LabMT, and an emoji sentiment score [22-24]. These 3 methods map a unigram of a word or emoji to a word score. ANEW consists of 3 scores per word, 1 relating to valence or happiness, 1 to arousal or excitement, and 1 relating to dominance or being influenced [22]. Similarly, LabMT is a measure of happiness, mapping words to a score for valence [23]. Finally, we used an emoji sentiment scale, which maps emojis, Unicode-based emoticons, to a happiness score [24]. After calculating the mean unigram score for the caption or comment section for each post, the average and SD for each unigram score were calculated as a feature.

### Data Postprocessing and Feature Extraction

In the postprocessing, individual profiles were filtered for quality and sufficient content. Following the guidelines of the CLPsych 2015 shared task, which asked participants to create methods for predicting depression in Twitter users, we restricted data from our original cohort of 2040 to individuals with at least 25 Instagram posts (removed n=755) and 75% English content (removed n=51), as measured by Google’s Compact Language Detector [25]. We further filtered the data by including only individuals with complete data in our dataset (removed n=482). The final included individuals have corresponding values for all variables, except for emoji scores, where a neutral value of “0” was imputed if the variable was missing. Finally, due to the small sample size, we only included male and female responders (removed n=3), for a total sample size of 749. The characteristics of the individuals in our cohort, including their extracted features, and the text-based scores are shown in Table 1. Compared with the initial cohort of 2040 individuals, there is no significant difference of the final cohort in gender proportion measured using a binomial test and in PHQ-8 scores as measured using a *t* test. However, there is a significant difference (*P*<.001) of ages that resulted from restricting our dataset to only active Instagram users, who are generally younger than the general population [9,10].

### Model Development

**Figure 1:**
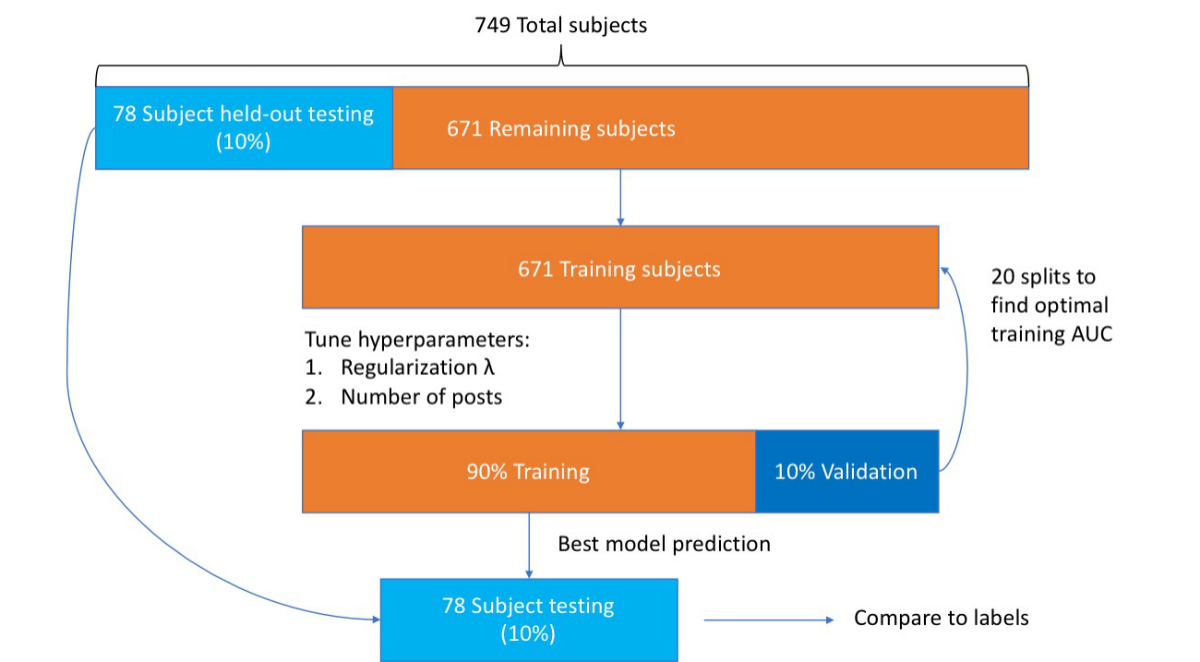
Overview of our machine learning methodology. From the original 749 participating individuals, 78 (ie, 10% of the dataset) were randomly selected and held out for testing. The remaining 671 cases were used for training and parameter-tuning through cross-validation. AUC: area under curve.

Figure 1 shows an overview of the machine learning methodology in this study. For all individuals, text-based features, including ANEW, LabMT, and emoji sentiment, were calculated from unigrams within texts of comments and captions to generate community-generated and user-generated features. The mean and SD of the text-based scores for the most recent *k* posts were utilized as features in our model training, with *k* as a hyperparameter tuned through cross-validation. We considered the summed PHQ-8 score as our target output and our extracted features as variables in a linear regression model, using an elastic-net regularization penalty to prevent overfitting.

To generate a test set to independently evaluate the performance of the model, 10% of the original 749 data points were randomly selected and excluded before training. For each *k* between 5 and 30, the training data were split into a 90/10 percentage training and validation set for 20 separate iterations. For each iteration, a linear regression model with an elastic-net regularization was fit to the sums of the PHQ-8 scores on the training data using the *glmnet* R package, whereas the results were evaluated on the internal validation data [26]. To find the optimal number of recent posts to use (*k*) and the regularization parameter (λ), the average validation area under curve (AUC) was calculated. This cross-validation found *k*=20 as the optimal value. We also used the median of the optimal λ for 20 iterations as a regularization parameter. In total, we trained 3 separate models: (1) based on only community-generated data, (2) based on only user-generated data, and (3) based on the combination of all variables from both sources. The discriminatory power of the generated models was compared on the held-out test set, using a binary indicator variable of depression as an input (ie, PHQ-8 ≥15). The AUC for all 3 models was calculated using the *ROCR*, *pROC*, and *verification* R packages [27,28].

**Table 1:** Our cohort characteristics and their associated features. The last column specifies which models, if any, contain the variable.

Characteristic			Statistics		Model inclusion (user/community)
Subjects (n)			749		Both
**Text-based scores, mean (SD)**					
	Emoji sentiment, captions	0.39 (0.25)		User-based	
	Emoji sentiment, comments	0.47 (0.17)		Community-based	
	ANEW^a^ valence, captions	6.55 (0.4)		User-based	
	SD ANEW valence, captions	1.05 (0.36)		User-based	
	ANEW domination, captions	5.66 (0.25)		User-based	
	SD ANEW domination, captions	0.66 (0.23)		User-based	
	ANEW arousal, captions	5.36 (0.25)		User-based	
	SD ANEW arousal, captions	0.65 (0.2)		User-based	
	LabMT^b^ score, captions	5.81 (0.23)		User-based	
	SD LabMT score, captions	0.57 (0.21)		User-based	
	ANEW valence, comments	6.83 (0.55)		Community-based	
	SD ANEW valence, comments	0.99 (0.5)		Community-based	
	ANEW domination, comments	5.77 (0.32)		Community-based	
	SD ANEW domination, comments	0.63 (0.3)		Community-based	
	ANEW arousal, comments	5.51 (0.3)		Community-based	
	SD ANEW arousal, comments	0.59 (0.23)		Community-based	
	LabMT score, comments	0.62 (0.29)		Community-based	
	SD LabMT score, comments	5.91 (0.34)		Community-based	
**Metadata, mean (SD)**					
	Number of posts	333.55 (476.59)		Both	
	Number of likes	27.25 (55.46)		Both	
	Number of comments per post	1.63 (1.8)		Both	
	Number of comments, total	245.25 (616.41)		Both	
	Fraction of posts with no captions	0.03 (0.07)		User-based	
	Fraction of posts with no comments	0.48 (0.24)		Community-based	
	Caption length by word	12.39 (10.07)		User-based	
	Comment length by word	10.09 (13.21)		Community-based	
**Demographics**					
	Age (years), mean (SD)	26.7 (7.29)		Neither	
	Female, n (%)	515 (68.8)		Both	
	Male, n (%)	234 (31.2)		Both	
	Asian, n (%)	51 (6.8)		Neither	
	Black, n (%)	143 (19.1)		Neither	
	Hispanic/Latino, n (%)	91 (12.1)		Neither	
	Native American/Alaskan Native, n (%)	10 (1.3)		Neither	
	Native Hawaiian/Pacific Islander, n (%)	2 (0.2)		Neither	
	Other, n (%)	27 (3.6)		Neither	
	White, n (%)	425 (56.7)		Neither	
**Depression**					
	PHQ-8^c^ score, mean (SD)	6.62 (5.22)		Neither	
	PHQ-8 ≥15, n (%)	69 (9.2)		Neither	

^a^ANEW: Affective Norms for English Words.

^b^LabMT: Language assessment by Mechanical Turk.

^c^PHQ-8: Patient Health Questionnaire-8.

## Results

### Outcome

**Figure 2:**
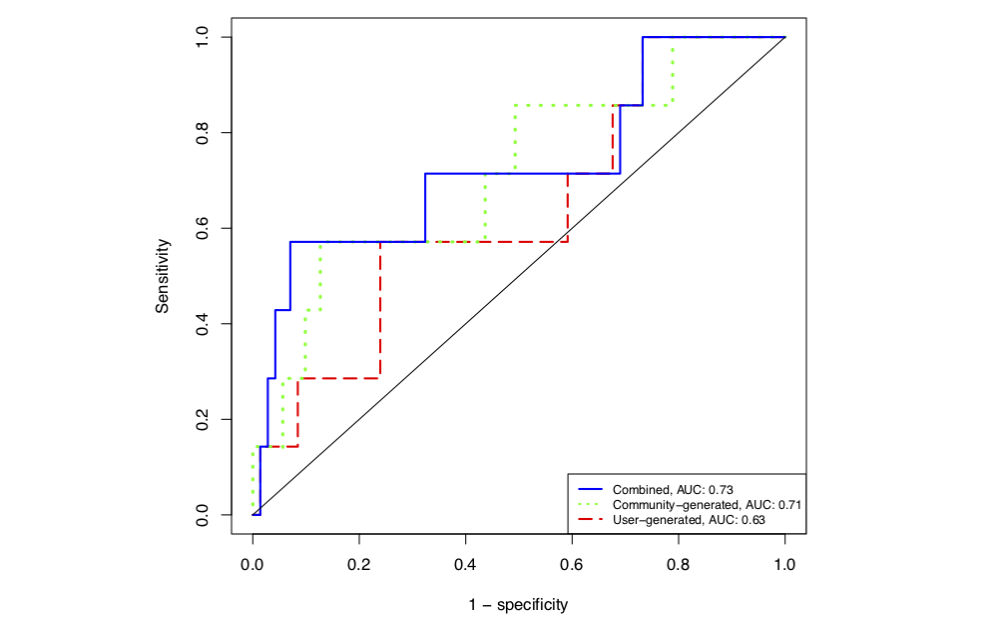
Classification receiver operating characteristic curves for the predictive capability of user-generated data, community-generated data, and the combination of both to predict major depressive disorder in 78 social media users. The models that included community-generated data were significantly better than random classification, as measured with a Mann-Whitney *U* test (*P*=.03 and *P*=.02 for community-generated and combined, respectively), whereas the model trained on only user-generated data was not (*P*=.11). AUC: area under curve.

The evaluation of the trained models on our held-out test set of 78 (10.4% (78/749) of the total dataset; in which 8 of the 78 had a PHQ-8 score at or above 15) indicated that community-generated data had significant predictive capacity for determining moderately severe to severe depression according to the PHQ-8 assessment (AUC=0.71; *P*=.03), whereas user-generated data were not significantly predictive (AUC=0.63; *P*=.11). When all features were combined to train a single, combined model, the model performed slightly better than the community-generated model (AUC=0.73; *P*=.02) alone, but this improvement was not statistically significant. Figure 2 shows the receiver operating characteristic curves of these 3 models on our independent test set.

**Figure 3:**
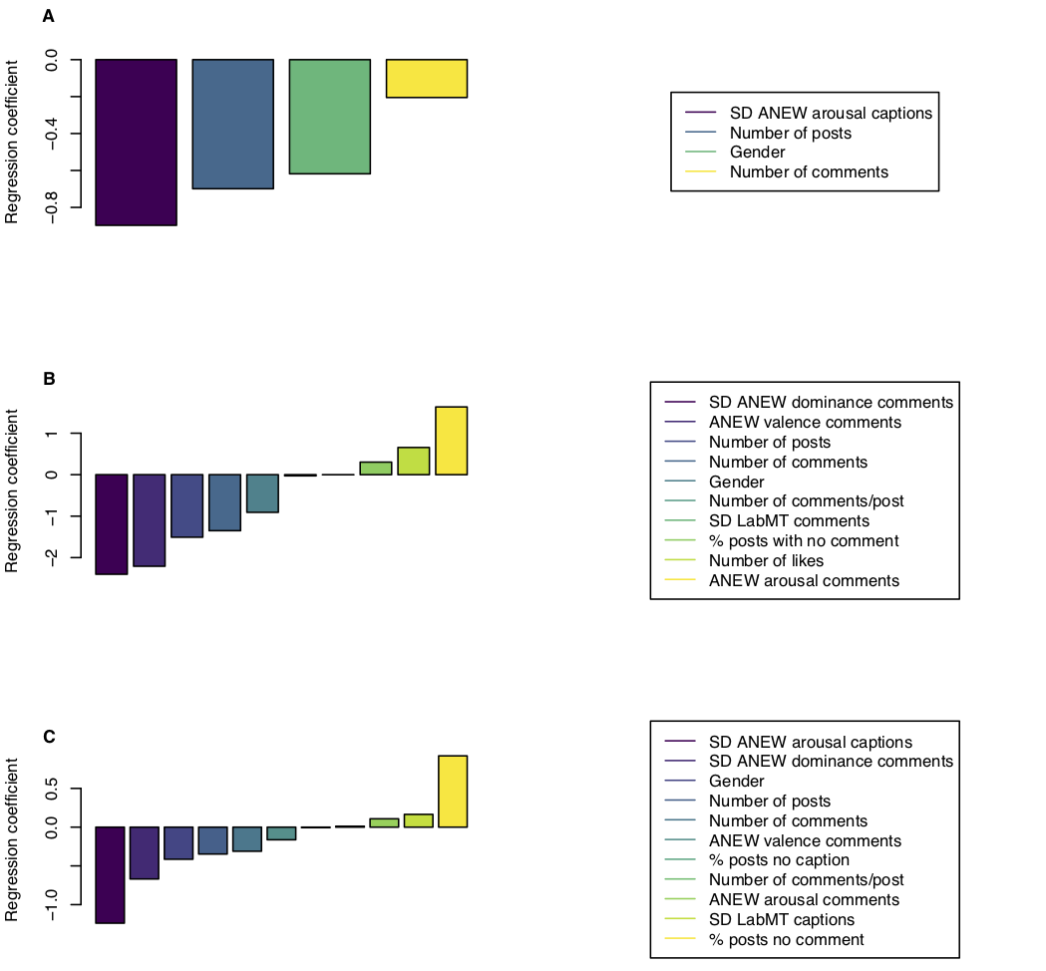
Minimum-maximum normalized linear regression coefficients for the model based on (A) user-generated data, (B) community-generated data, and (C) both. “Gender” variable indicates if the individual is male. These weights indicate the relative importance of each feature in the corresponding model. ANEW: Affective Norms for English Words; LabMT: Language assessment by Mechanical Turk.

Our sensitivity analysis showed that at different values of *k*, the cutoff for the most recent posts, the model based on the combination of community-generated and user-generated data still outperforms the other 2 models. To understand the composition of the model, we utilized the linear regression weights of minimum-maximum normalized variables to identify the indicative features in each model (see Figure 3 and Multimedia Appendix 3).

### Outcome

Importantly, the model combining the 2 different feature sets did not simply use the community-generated or user-generated data alone. The highest corresponding weights in this combined model were features extracted both from user-generated and community-generated data; the SD of ANEW arousal caption scores, and the SD of ANEW dominance comment scores, respectively, as opposed to only using information from either dataset individually. Furthermore, the other influential variables also consisted of a combination of user-generated variables (SD of ANEW arousal caption scores, percentage of posts without captions, and SD of LabMT caption scores) and community-generated variables (SD of ANEW dominance comment scores, percentage of posts without comments, and ANEW valence comment scores).

To identify the influence on the number of recent posts on our models, we performed a sensitivity analysis by adjusting different numbers of recent posts *k* for each model. This analysis revealed that decreasing the number of incorporated posts resulted in user-generated data becoming more informative than comments in terms of predicting MDD. Conversely, comments became more informative than captions with the inclusion of more posts. However, the combination of both consistently outperformed either user-generated or community-generated data alone, indicating that community-generated data contain vital information on mental health status that is not captured within user-generated data alone.

## Discussion

### Principal Findings

This study explored the utility of community-generated social media data for identifying depression among social media users. The results indicate that community-generated social media content contains information indicative of a social media user’s depression and that a model trained on the combination of user-generated and community-generated social media data outperforms models using either data source alone. Further analysis of the resulting models reveals that the indicative features from community-generated and user-generated data for this task are largely complementary and nonoverlapping.

### Using Community-Generated Data Improves Detection of Depression

To the best of our knowledge, the study presented in this paper shows for the first time that information extracted from community-generated content on social media, specifically “post comments,” can be used to identify mental illness in individuals with similar capacity as user-generated data. Although previous work has incorporated community-generated metadata, such as number of comments, much of the previous research has largely been focused on understanding a user’s mental well-being through information that the user posts on social media, such as Twitter “Tweets,” Facebook status updates, or Instagram images [13-15,21]. Other studies also have suggested that community-generated data are correlated with user-generated data, as alcohol-related posts have more positive community-generated data [29]. Our results presented in this paper add to the body of evidence that inclusion of community-generated data may benefit analysis of social media users.

In this study, we showed that data generated from the interaction of other users with an individual carry information about a clinically validated depression assessment (PHQ-8). The model trained on community-generated data classifies individuals with a PHQ-8 score ≥15 significantly better than random classification, whereas the model trained with user-generated data did not perform as well. The model using the combination of both community-generated and user-generated datasets outperformed both; however, the improvement of this model upon the community-generated model is not statistically significant. These results indicate that future research may benefit from incorporating community-generated data, in addition to user-generated data, to understand and predict mental health in social media users.

To determine the potential for our models to be used for clinical purposes, model performance characteristics were calculated for optimal threshold values. Our model’s results for detecting depression are comparable to unaided physician performance [30], demonstrating the potential for community-generated content to be implemented into screening populations for MDD. These results are not meant to be directly comparable, due to differences in population and methodology between our study and the meta-analysis performed by Mitchell et al [30]. However, the results suggest that the use of community-generated data can be beneficial for mental health screening and can be improved to the point of clinical relevance.

### Community-Generated Data Contain Unique Information Not Captured in User-Generated Data

A concern regarding the combined model is the distribution of its indicative features among user-generated and community-generated variables and whether community-generated data and user-generated data provide similar information or a high degree of correlation, which would limit the utility of including community-generated data in future research. To analyze the variable distribution and their overlap among different models, the minimum-maximum normalized variable weights in each of the models were examined. This normalization allows a direct comparison of the feature coefficients within each model by rescaling all variables between “0” and “1.”

These model weights indicate that for the model using both user-generated and community-generated data, the extracted features are considered informative, indicating that user-generated and community-generated data contain unique, complementary information and are nondegenerate. Furthermore, in all 3 models, there were variables that were given more weight than gender, a variable consistently shown to be attributed to different rates of depression, with women having a higher predisposition to depression [31-33]. This indicates the utility of social media–based features, both community-generated and user-generated, as an informative source for detecting depression, in addition to previously explored demographic information.

Surprisingly, the user-generated model did not perform as well as the community-generated model. A potential explanation concerns the lack of time data. The model presented was optimized to prioritize comment data over user-generated data, particularly in choosing the number *k* of recent posts to use. Sensitivity analysis indicated that captions generally performed better with fewer recent posts used and comments performed better with more recent posts used. A user may have a more variable mood through timeline given for a series of posts, and potentially, the community may provide a more stable signal over a longer period. However, the combination of features consistently outperformed either when used alone.

### Comparison With Previous Work

Prediction of MDD based on social media data is well established with strong results [13-17,21,34]. However, the existing literature has largely focused on using user-generated data for this purpose, with minimal amount of community-generated data analysis. This study demonstrates that community-generated content contains information complementary to user-generated data, which can be used to predict MDD in a given user. In particular, these results suggest that community-generated text (eg, “comments”) may be useful for predicting MDD, as opposed to only network/graph type features (eg, “followers”) currently used in research [17].

In previous work on predicting MDD based on user-generated data, a random forest model trained on user-generated Twitter data showed promising results for predicting depression [14]. Our study had a significantly larger dataset, with 749 total individuals compared with 204, from a different social media platform (Instagram). The features incorporated in our models were partially inspired by the variables in this study, which included ANEW and LabMT scores, as well as word counts. In future work, we plan to improve the presented models through incorporating data-driven feature extraction, instead of a priori feature selection.

**Table 2:** Optimal cutoffs using the highest observed *F* score for user-generated, community-generated, combined, and bag-of-words models and comparison with physician rates.

Method	*F*_*1*_ score	Sensitivity	Specificity
Physician (meta-analysis [30])	0.62	0.50	0.81
Baseline feature set (BOW^a^)	0.58	0.50	0.69
User-generated	0.66	0.57	0.77
Community-generated	0.69	0.57	0.87
Community- and user-generated	0.70	0.57	0.92

^a^BOW: bag-of-words.

### Potential for Clinical Use

A prior meta-analysis of 118 studies indicated that physicians, without the use of scales or other diagnostic tools, had an average sensitivity of 50% and a specificity of 81% for detecting depression [30]. At respective optimal cutoffs, our models had a sensitivity of 57% and specificities of 0.76, 0.86, and 0.93 for user-generated data, community-generated data, and the combination of both, respectively (Table 2). This analysis indicates the potential of community-generated data alone to diagnose moderately severe to severe depression at levels comparable to physician diagnosis. The model’s performance further improves with the addition of user-generated data. It is important to note that the population and methodology used for the meta-analysis are fundamentally different from the research presented here, and interpretations between the 2 studies should be performed with caution.

To evaluate the use of a baseline feature set, a bag-of-words (BOW) model was used. Each post’s caption and comment were tokenized, and English stop words were removed using the Natural Language Tool Kit library [35]. The processed captions and comments of a user’s most recent 20 posts were aggregated and converted to a feature vector according to the BOW model. A regularized linear regression was trained based on these feature vectors according to the same procedure applied to the previously generated models, and its performance was compared with the previously presented models in this paper (Table 2).

The results indicate that this baseline model does not perform as well as the other presented models. This low performance can be due to the smaller sample size in this study and the simplicity and sparsity of the features in the BOW model. The features in this baseline model only rely on the frequency of words and do not capture explicit information about the word semantics and sentiment. In addition, many captions and comments are short, with an average of 12.39 and 10.09 words, respectively, in our dataset. Of note, the number of features (ie, the number of unique words) in the BOW model was 49,497 for 671 training samples, which contributed to the feature sparsity in the baseline model.

This method may also potentially be used as a cost-effective metric for the evaluation of interventions. Similar to approaches analyzing the effectiveness of other behavioral or pharmacological interventions, this method could be used as a low-cost means of patient monitoring. This is especially valuable among youth and adolescent populations, who tend to display less compliance with ecological momentary assessment reporting [36,37].

### Limitations and Future Work

Due to our deanonymization protocol, time stamps, in addition to other identifiers, were removed from posts in our dataset. Therefore, we only had access to the chronological order of the posts rather than their exact time stamps. Other studies have used time series and chronologically dependent variables to understand depression in social media users [14,16]. Such analysis was not possible in this project. The PHQ-8 questionnaire represents a timeline of the previous 2 weeks; however, one of the shortcomings of utilizing the most recent *k* posts is that these *k* posts may not fully represent the posts in the last 2-week period. Future studies should incorporate time data to potentially improve outcomes. Another current limitation is that the comment section may contain some user-generated information, specifically comments generated by the user themselves. These user-generated comments could not be recognized and removed in our current dataset due to our deanonymization protocol of removing user identification. However, a significant portion of the comments is not generated by the user themselves, and most information comes from extrinsically defined sources. Finally, we acknowledge the relatively small sample size of MDD-positive individuals in our testing set (8 of 78); however, the statistical hypothesis test determining the presence of non-null significant difference in ranks between MDD-positive and -negative individuals considers sample size intrinsically in *P* values generated at the 95% confidence level. Leveraging a larger dataset with data-driven feature selection in future work can improve the training of models.

### Conclusions

Social media content has been utilized previously to identify depression; however, much research to date has focused mostly on the information that individuals generate as opposed to content generated by other users, such as comments or “likes.” The results presented in this paper indicate that data generated from persons who interact with posts made by other social media users contain information about the mental health of those users, specifically depression status. Furthermore, this study found that community-generated data are complementary and nonoverlapping, with respect to the content generated by the user themselves.

## Appendix

Multimedia Appendix 1 Example Instagram post. Each post consists of an image or a video, with an optional caption generated by the user. Friends or followers of the user can “Like” or comment on the photo.

Multimedia Appendix 2 Patient Health Questionnaire-8. For each question, responders are asked for the number of days they have been affected by each symptom. The numeric responses are summed for a response. In this study, a score at or above 15 is considered positive for major depressive disorder.

Multimedia Appendix 3 Coefficients of models based on user-generated, community-generated, and combined data.

## Abbreviations

ANEW: Affective Norms for English Words
AUC: area under curve
BOW: bag-of-words
LabMT: Language assessment by Mechanical Turk
MDD: major depressive disorder
PHQ-8: Patient Health Questionnaire-8
