# Supplementary figures and images for: Exploring the Utility of Community-Generated Social Media Content for Detecting Depression: An Analytical Study on Instagram
Source: J Med Internet Res. 2018 Dec 6;20(12):e11817. doi: 10.2196/11817 (PMC6302231; doi:10.2196/11817)

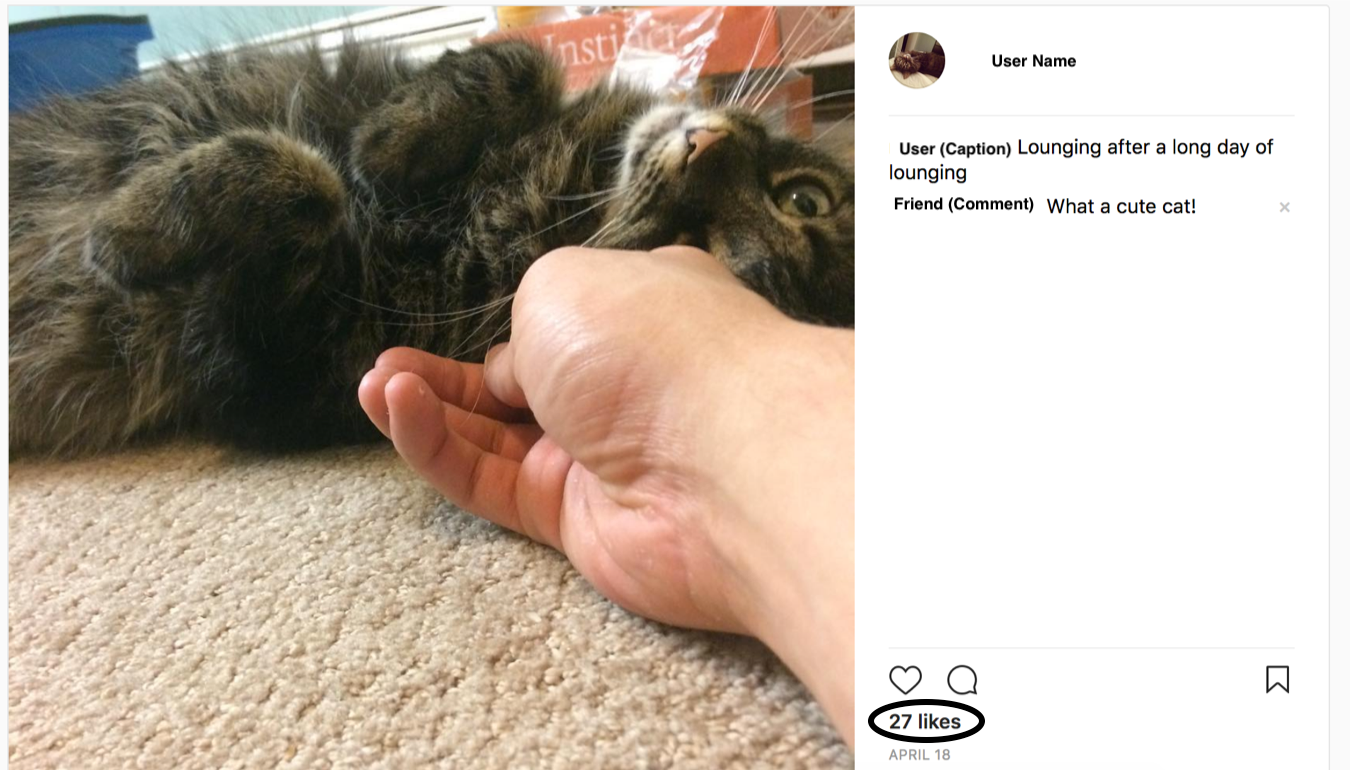

Supplement: Multimedia Appendix 1 [file jmir_v20i12e11817_app1.png]
