# Supplementary material for: Exploring the Utility of Community-Generated Social Media Content for Detecting Depression: An Analytical Study on Instagram
Source: J Med Internet Res. 2018 Dec 6;20(12):e11817. doi: 10.2196/11817 (PMC6302231; doi:10.2196/11817)
Supplement: Multimedia Appendix 2 [file jmir_v20i12e11817_app2.pdf]

Table S1. PHQ8 questionnaire. For each question, responders are asked for the number of days they have been affected by each symptom. The numeric responses are summed for a response. In this study, a score at or above 15 is considered positive for MDD.

How often during the past 2 weeks were you bothered by…	Not at all (0)	Several days (1)	More than half the days (2)	Nearly every day (3)
Little interest or pleasure in doing things				
Feeling down, depressed, or hopeless				
Trouble falling or staying asleep, or sleeping too much				
Feeling tire or having little energy				
Poor appetite or overeating				
Feeling bad about yourself, or that you are a failure, or have let yourself or your family down				
Trouble concentrating on things, such as reading the newspaper or watching television				
Moving or speaking so slowly that other people could have noticed. Or the opposite - being so fidgety or restless that you have been moving around a lot more than usual				
