# Supplementary material for: Exploring the Utility of Community-Generated Social Media Content for Detecting Depression: An Analytical Study on Instagram
Source: J Med Internet Res. 2018 Dec 6;20(12):e11817. doi: 10.2196/11817 (PMC6302231; doi:10.2196/11817)
Supplement: Multimedia Appendix 3 [file jmir_v20i12e11817_app3.pdf]

Table S2. Coefficients of models based on user-generated, community-generated, and combined data.

Model	Coefficient/Weight
**User-Generated**	
Intercept	7.816
Sd. Dev. ANEW Arousal, Captions	-0.897
Number of Posts	-0.699
Gender	-0.618
Number of Comments, total	-0.205
**Community-Generated**	
Intercept	8.736
Sd. Dev. ANEW Domination, Comments	-2.401
Average ANEW Valence, Comments	-2.208
Number of Posts	-1.508
Number of Comments, total	-1.351
Gender	-0.908
Number of Comments per Post	-0.0018
Sd. Dev. LabMT Comments	0.028
% Posts with No Comments	0.303
Number of Likes	0.656
Average ANEW Arousal Comments	1.634
**Combined (User- and Community-Generated)**	
Intercept	8.302
Sd. Dev. ANEW Arousal, Captions	-1.239
Sd. Dev. ANEW Domination, Comments	-0.920
Gender	-0.669
Number of Posts	-0.414
Number of Comments, total	-0.347
Average ANEW Valence, Comments	-0.311
% Posts with no Captions	-0.163
Number of Comments per Post	-0.007
Average ANEW Arousal, Comments	0.012
Sd. Dev LabMT Captions	0.108
% Posts with No Comments	0.165
